# Outcome-specific shifts in homocysteine-androgen correlations in polycystic ovary syndrome

**DOI:** 10.3389/fendo.2026.1809890

**Published:** 2026-04-07

**Authors:** Qian Han, Jiarun Zhao, Mengyuan Li, Mingwei Xin, Xiaodan Yin, Junqin He

**Affiliations:** 1 Department of Traditional Chinese Medicine, Beijing Obstetrics and Gynecology Hospital, Capital Medical University, Beijing Maternal and Child Health Care Hospital, Beijing, China; 2 Capital Medical University, Beijing, China

**Keywords:** androgens, homocysteine, metabolic heterogeneity, polycystic ovary syndrome, pregnancy outcomes

## Abstract

**Background:**

Elevated homocysteine (HCY) and hyperandrogenism are key metabolic disturbances in polycystic ovary syndrome (PCOS), but their relationship across different reproductive outcomes remains unclear.

**Objective:**

To investigate outcome-specific associations between HCY and androgen profiles in PCOS women with infertility, live birth, or spontaneous abortion.

**Methods:**

This retrospective study enrolled 875 PCOS patients, classified into infertility (n=302) and pregnancy (n=573) groups. After excluding non-spontaneous pregnancy losses, the pregnancy group was further subdivided into live birth (n=266) and spontaneous abortion (n=292). Spearman correlation and multivariable linear regression were used to assess HCY-androgen associations.

**Results:**

Metabolic disturbances were most pronounced in infertile patients, followed by the spontaneous abortion group, while the live birth group exhibited the most favorable metabolic profile (all P < 0.05). HCY-androgen correlations varied markedly across reproductive outcomes. In infertile patients, HCY showed broad positive correlations with multiple androgens, with regression analysis confirming independent associations with total testosterone (TT), bioavailable testosterone (Bio-T), free testosterone (FT) and free androgen index (FAI) (β = 0.12–0.21, all P < 0.01). These correlations narrowed substantially in pregnant women. Strikingly, a divergent pattern emerged within pregnancy subgroups: HCY was independently positively associated with TT in the live birth group (β = 0.30, P < 0.01), but independently negatively associated with dehydroepiandrosterone (DHEA) in the spontaneous abortion group (β = -0.16, P = 0.02).

**Conclusion:**

The HCY-androgen associations in PCOS are reproductive outcome-specific and may inform risk stratification across different reproductive stages.

## 1 Introduction

Polycystic ovary syndrome (PCOS) is the most common endocrine and metabolic disorder affecting women of reproductive age. Its classic clinical features include hyperandrogenism, insulin resistance, ovulatory dysfunction, and polycystic ovarian morphology (1). With the continuous evolution of diagnostic criteria, the epidemiological landscape of PCOS has been progressively updated. A recent 2026 meta-analysis reported that, according to the Rotterdam criteria, the global prevalence of PCOS among adult women has reached 12.1% (2). The syndrome is not only characterized by menstrual irregularities, hirsutism, and acne, but also represents a leading cause of female infertility and miscarriage, while significantly increasing the risk of severe pregnancy complications such as gestational diabetes mellitus and preeclampsia (3). Reproductive function is markedly compromised in PCOS, with the condition accounting for up to 80% of cases of anovulatory infertility (4). Multiple mechanisms—including impaired endometrial receptivity, chronic low-grade inflammation, and oxidative stress imbalance—are involved in the pathological processes underlying PCOS-related infertility and pregnancy loss (5, 6).

Hyperandrogenism, a central feature of PCOS, has been increasingly recognized as a critical contributor to the development and progression of reproductive dysfunction in this population (7). PCOS patients with a hyperandrogenic phenotype are more susceptible to ovulatory disorders and diminished endometrial receptivity, thereby facing significantly elevated risks of adverse pregnancy outcomes such as miscarriage and preterm birth (8, 9). Of note, hyperandrogenism does not operate in isolation; rather, it engages in complex interactions with other pathological states—including insulin resistance and coagulation abnormalities—that collectively influence pregnancy outcomes in PCOS (10–12). However, clinical observations reveal that not all hyperandrogenic PCOS patients experience adverse pregnancy outcomes, and conversely, some patients without overt hyperandrogenism still suffer from such events. This clinical paradox suggests the existence of additional key modulators in the pathophysiology underlying PCOS-related pregnancy outcomes, which warrant further investigation.

Homocysteine (HCY), a sulfur-containing amino acid involved in one-carbon metabolism, has been implicated in the pathophysiology of PCOS (13, 14). Elevated HCY levels are frequently observed in PCOS patients (15), and exogenous HCY administration has been shown to induce PCOS-like phenotypes in murine models, characterized by dysregulated glucolipid metabolism and hyperandrogenism (16). HCY contributes to the development of hyperandrogenism through mechanisms involving upregulation of steroidogenic acute regulatory protein and inhibition of aromatase activity (16). Moreover, HCY may disrupt the reproductive microenvironment and compromise pregnancy outcomes by interfering with androgen metabolism, glucolipid homeostasis, and inflammatory responses. Notably, to date, no study has systematically investigated whether the pattern of HCY-androgen associations differs across PCOS subpopulations stratified by distinct reproductive outcomes—specifically infertility, live birth, and spontaneous abortion. Elucidating these outcome-specific correlation patterns may provide novel insights into the heterogeneity of reproductive outcomes in PCOS.

The present study conducted a retrospective cohort analysis aimed at comparing the differential associations between HCY and androgen profiles in PCOS patients stratified by reproductive outcomes—specifically infertility, pregnancy, live birth, and spontaneous abortion. By elucidating the dynamic correlation patterns between HCY and androgens across these distinct clinical endpoints, this study seeks to provide novel clinical evidence contributing to a more nuanced understanding of the heterogeneity in reproductive outcomes among women with PCOS.

## 2 Materials and methods

### 2.1 Study population

This retrospective cohort study was approved by the Medical Ethics Committee of Beijing Obstetrics and Gynecology Hospital, Capital Medical University (Approval No. 2025-KY-047-01). We enrolled PCOS patients with fertility aspirations who attended Beijing Obstetrics and Gynecology Hospital, Capital Medical University between June 2019 and April 2025.

Inclusion criteria were: (1) diagnosis of PCOS based on the 2003 Rotterdam criteria; and (2) availability of complete endocrine and metabolic data relevant to this study. Exclusion criteria included the presence of organic or structural abnormalities of the reproductive tract, such as endometriosis, uterine fibroids, endometrial polyps, and uterine malformations. Patients with endocrine disorders—including hyperthyroidism and adrenal dysfunction—were also excluded, as were those with severe cardiovascular, hepatic, renal, or cerebrovascular diseases requiring concomitant treatment with antihypertensive agents, glucocorticoids, or other medications.

Patients were stratified into an infertility group and a pregnancy group according to whether pregnancy was achieved. To further investigate differences across pregnancy outcomes, a subgroup analysis was conducted among pregnant patients. After excluding cases of pregnancy termination due to ectopic pregnancy, gestational trophoblastic disease, fetal malformations, or induced labor, the remaining patients were divided into live birth and spontaneous abortion groups.

Infertility was defined as failure to conceive after at least 12 months of regular, unprotected intercourse. Pregnancy was defined as serum β-Human Chorionic Gonadotropin(β-HCG) >5 IU/L. Live birth was defined as delivery of a viable infant. Spontaneous abortion was defined as pregnancy loss before 28 weeks of gestation.

### 2.2 Data collection

Baseline characteristics, including age, height, weight, menstrual status, and reproductive history, were collected from all participants. Laboratory data comprised serum androgen profiles (Total Testosterone(TT), Bioavailable Testosterone(Bio-T), Androstenedione(A2), Dihydrotestosterone(DHT), Free Testosterone(FT), Free androgen index(FAI), Dehydroepiandrosterone(DHEA), Dehydroepiandrosterone Sulfate(DHEAS), 17-Hydroxyprogesterone(17-OHP)), Sex Hormone Binding Globulin(SHBG), anti-Müllerian hormone(AMH), follicle-stimulating hormone(FSH), luteinizing hormone(LH), fasting glucose, fasting insulin, platelet count(PLT), and HCY. FSH and LH were measured in blood samples obtained during days 2–5 of the menstrual cycle.

Androgen profiling was performed using liquid chromatography-tandem mass spectrometry (LC-MS/MS), a technique that optimizes the diagnosis of hyperandrogenism and enables more precise identification of adverse reproductive risk in PCOS (17). This LC-MS/MS method for androgen profiling in PCOS was previously established by our institution with rigorous quality control, including assessments of linearity, lower limit of quantitation, precision, accuracy, matrix effect, and serum sample stability (18, 19). TT represents the sum of all forms of testosterone in serum. Bio-T refers to the bioactive testosterone that can exert biological effects *in vivo*. A2 is a weak androgen originating from both the ovaries and adrenal glands. DHT is a potent androgen with high binding affinity for the androgen receptor. FT refers to the fraction of testosterone not bound to any protein, which can directly enter target cells and exert its effects. FAI indirectly reflects free testosterone levels. DHEA and DHEAS are androgens primarily derived from the adrenal glands, with a minor contribution from the ovaries. 17-OHP is an important intermediate in the synthesis of cortisol by the adrenal cortex and is used to rule out congenital adrenal hyperplasia.

Insulin resistance was assessed using the homeostatic model assessment for insulin resistance (HOMA-IR), calculated as: HOMA-IR = fasting glucose (mmol/L) × fasting insulin (μU/mL)/22.5 (20).

The FAI was calculated as: FAI = TT (nmol/L)/SHBG (nmol/L) × 100% (21).

### 2.3 Statistical analysis

Statistical analyses were performed using SPSS version 27.0. Normality of data distribution was assessed using the Kolmogorov–Smirnov test. Continuous variables are expressed as mean ± SD for normally distributed data, or as median (interquartile range, IQR) for non-normally distributed data. Between-group comparisons were conducted using the independent t-test (for normally distributed variables) or the Mann–Whitney U test (for non-normally distributed variables). Spearman’s rank correlation was used to evaluate the associations between HCY levels and androgen parameters. Multivariable linear regression models were constructed to assess the independent associations between HCY and androgens, with adjustment for age, BMI, platelet count, HOMA-IR, AMH, and LH/FSH ratio, which showed baseline differences. Multicollinearity among variables was assessed using VIF. High multicollinearity (VIF > 5) was observed among TT, FT, Bio-T, and FAI. To avoid interference with regression coefficient estimation due to collinearity, these four variables were incorporated separately into four independent linear regression models. Each model was constructed including non-collinear indicators (SHBG, A2, 17-OHP, DHT, DHEAS, DHEA). All statistical tests were two-tailed, and P < 0.05 was considered statistically significant.

## 3 Results

### 3.1 Study population screening process

**Figure 1:**
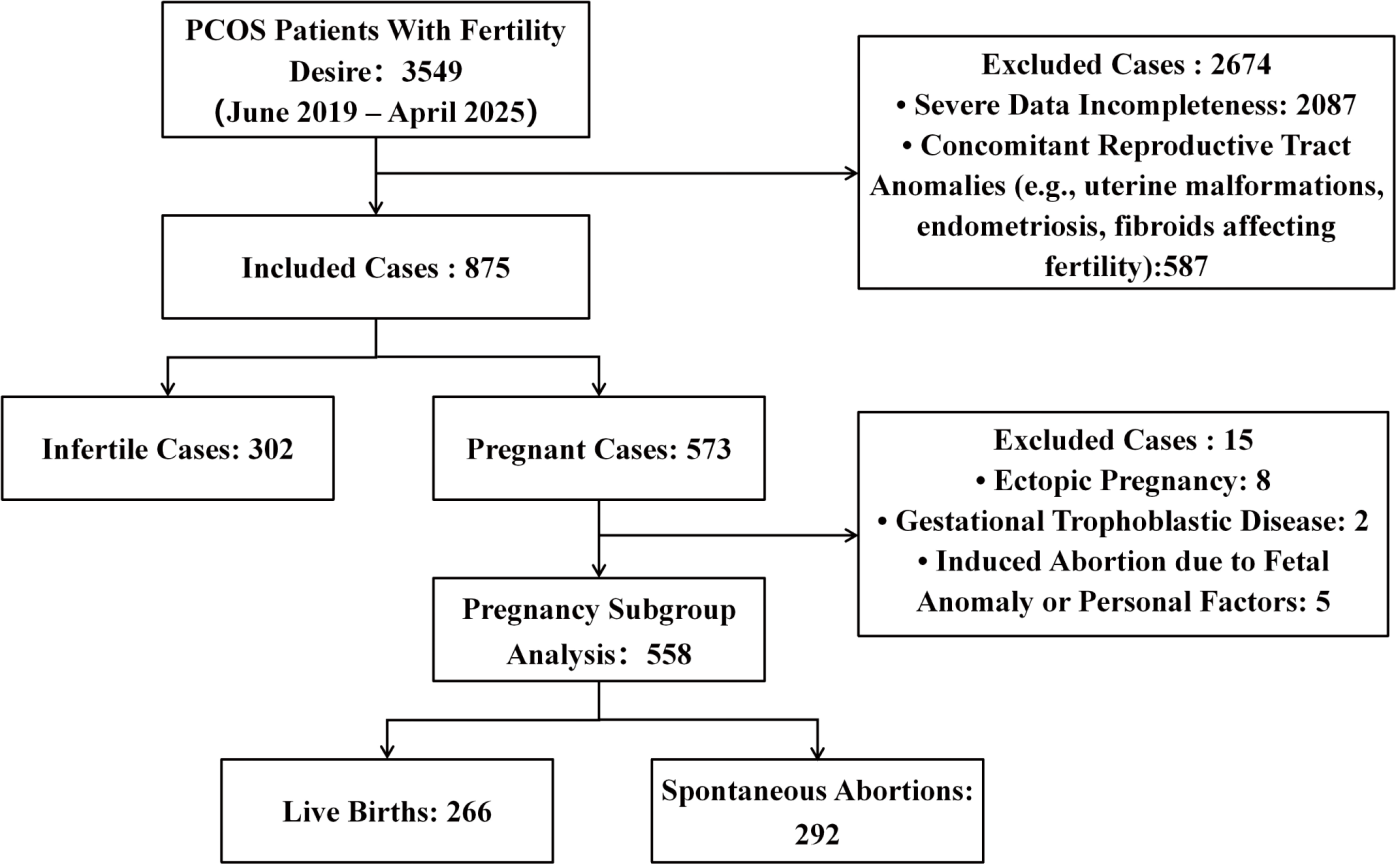
Study flowchart.

A total of 3,549 patients with PCOS were initially screened. After rigorous application of the inclusion and exclusion criteria, 875 eligible patients were ultimately enrolled for statistical analysis, comprising 302 patients in the infertility group and 573 in the pregnancy group. Within the pregnancy group, 15 cases of pregnancy loss due to non-spontaneous causes—including ectopic pregnancy, gestational trophoblastic disease, and fetal developmental abnormalities—were excluded. The final pregnancy subgroup analysis included 558 patients, consisting of 266 in the live birth group and 292 in the spontaneous abortion group (including 288 cases of early miscarriage and 4 cases of late miscarriage) (**Figure 1**).

### 3.2 Analysis of infertile and pregnant patients with PCOS

#### 3.2.1 Baseline characteristics of infertile and pregnant patients

PCOS patients in the infertility and pregnancy groups exhibited significant phenotypic differences (**Table 1**). Compared with the pregnancy group, patients in the infertility group were younger and had lower SHBG levels, while presenting with higher levels of Body mass index(BMI), AMH, LH/FSH ratio, TT, Bio-T, A2, DHT, FT, FAI, DHEA, HOMA-IR, HCY, and platelet count (all P < 0.05). These findings indicated that infertile PCOS patients manifest more pronounced endocrine disturbances and metabolic abnormalities.

**Table 1:** Baseline characteristics of PCOS-I and PCOS-P groups.

Characteristics	PCOS-P (n=573)	PCOS-I (n=302)	*Z/t*	*P* value
Age(years)	32(29, 34)	29(26, 33)	-6.73	<0.01^**^
BMI(kg/cm^2^)	24.46(21.87, 27.98)	25.12(22.04, 29.10)	-2.27	0.02^*^
AMH(ng/mL)	6.45(4.05, 9.15)	7.16(5.12, 11.15)	-3.66	<0.01^**^
LH/FSH	1.52 ± 0.93	1.78 ± 0.96	0.38	<0.01^**^
SHBG(nmol/L)	40.40(25.20, 70.0)	31.25(20.77, 60.40)	-3.77	<0.01^**^
Bio-T(pg/mL)	117.50(77.22, 190.17)	158.00(94.78, 223.00)	-5.34	<0.01^**^
A2(ng/mL)	1.54 ± 0.83	1.77 ± 0.73	0.69	<0.01^**^
17-OHP(ng/mL)	0.49(0.29, 0.79)	0.49(0.34,0.76)	-0.40	0.69
DHT(ng/mL)	0.16 ± 0.07	0.18 ± 0.06	0.06	<0.01^**^
FT(pg/mL)	4.73(3.07, 7.36)	6.36(3.87,8.81)	-5.48	<0.01^**^
TT(nmol/L)	1.22 ± 0.65	1.43 ± 1.26	0.10	<0.01^**^
FAI	0.03(0.01, 0.05)	0.036(0.02,0.06)	-5.31	<0.01^**^
DHEAS(ng/mL)	1825.39(1252.59,2461.65)	1964.43(1289.36,2711.90)	-1.95	0.05
DHEA(ng/mL)	3.05(1.85,4.86)	3.78(2.46,6.15)	-4.33	<0.01^**^
HOMA-IR	2.92(1.84, 4.04)	3.58(2.24,6.16)	-6.10	<0.01^**^
HCY(umol/L)	7.30(6.30, 8.90)	9.30(7.90,11.30)	-6.23	<0.01^**^
PLT(*10^9^/L)	273.50 ± 60.70	292.80 ± 59.70	0.98	<0.01^**^

^*^P<0.05; ^**^P<0.01; PCOS-P, PCOS with Pregnancy; PCOS-I, PCOS with Infertility.

#### 3.2.2 Correlation analysis of endocrine and metabolic parameters in infertile and pregnant patients

**Figure 2:**
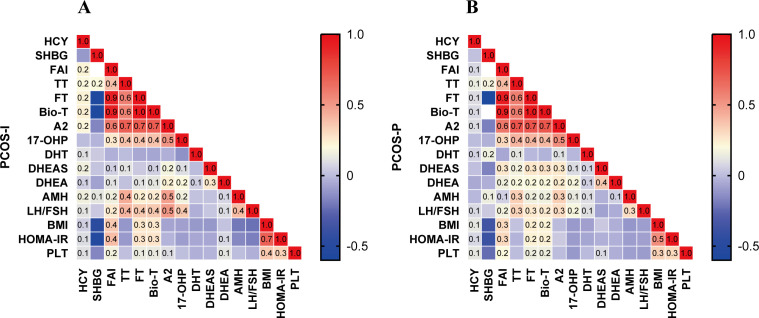
Correlation heatmap of endocrine and metabolic indices in PCOS-I and PCOS-P. **(A)** PCOS-I,Infertile group; **(B)** PCOS-P,Pregnant group. Values in each cell represent Pearson’s correlation coefficients (*r*). The color bar indicates the strength and direction of correlation, with red denoting positive correlations, blue denoting negative correlations, and color intensity proportional to the magnitude of *r*.

To further investigate the correlations between HCY and androgens in infertile and pregnant patients, Spearman correlation analysis was performed. As shown in **Figure 2**, strong positive correlations were observed within the androgen metabolic pathway in both groups, and AMH was significantly correlated with TT, A2, Bio-T, and FT, consistent with previous studies (19).

In infertile patients, HCY demonstrated strong positive correlations with TT, Bio-T, A2, FT, FAI, and DHEAS (all P < 0.05) (**Figure 2A**). In pregnant patients, the correlations between HCY and androgens were attenuated, with significant but weaker correlations observed only with TT, A2, and FT (P < 0.05) (**Figure 2B**). Significant differences in correlation coefficients between the two groups were observed for HCY with Bio-T, A2, FT, FAI, and DHEAS (all P < 0.05) (**Supplementary Table S1**).

#### 3.2.3 Multivariable linear regression analysis of HCY and androgens in infertile and pregnant patients

In multivariable linear regression models adjusted for age, AMH, LH/FSH ratio, platelet count, and HOMA-IR, HCY remained significantly and positively associated with TT, Bio-T, FT, and FAI in infertile patients (all P < 0.01). Specifically, each one-unit increase in TT, Bio-T, FT, and FAI corresponded to an increase in HCY levels of 0.21, 0.20, 0.20, and 0.20 units, respectively. In pregnant patients, the positive associations between HCY and androgens were attenuated; HCY was positively associated with TT and negatively associated with DHEA. Specifically, each one-unit increase in TT was associated with a 0.208-unit increase in HCY, whereas each one-unit increase in DHEA was associated with a 0.119-unit decrease in HCY (**Table 2**).

**Table 2:** Multivariable linear regression analysis of HCY and androgens in PCOS-I and PCOS-P groups.

Variables	PCOS-I		PCOS-P	
	*β*	*P*	*β*	*P*
SHBG	-0.05	0.45	-0.03	0.47
Bio-T	0.20	<0.01^**^	0.09	0.18
A2	0.02	0.78	-0.04	0.54
17-OHP	-0.06	0.30	-0.09	0.08
DHT	0.06	0.27	0.04	0.31
FT	0.20	<0.01^**^	0.09	0.14
TT	0.21	<0.01^**^	0.21	<0.01^**^
FAI	0.20	<0.01^**^	0.07	0.24
DHEAS	0.06	0.32	0.03	0.57
DHEA	0.06	0.30	-0.12	<0.01^**^

^**^P<0.01; PCOS-P, PCOS with Pregnancy; PCOS-I, PCOS with Infertility. The coefficients of non-collinear variables were derived from the model including TT. These coefficients showed minimal variation across the four models, and the consistency of P-values remained unchanged, indicating model robustness.

### 3.3 Analysis of PCOS patients with live birth and spontaneous abortion

#### 3.3.1 Baseline characteristics of patients with live birth and spontaneous abortion

Subgroup analysis was performed by further dividing the PCOS pregnancy cohort into live birth and spontaneous abortion groups, which demonstrated significant differences in baseline characteristics (**Table 3**). Compared with the live birth group, patients in the spontaneous abortion group exhibited lower SHBG levels, along with significantly higher BMI, HOMA-IR, TT, Bio-T, A2, FT, FAI, HCY, and platelet count (all P < 0.05). These findings indicated that PCOS patients who experience spontaneous abortion present with more pronounced endocrine disturbances and metabolic abnormalities than those who achieve live birth.

**Table 3:** Baseline characteristics of PCOS-AB and PCOS-L groups.

Characteristics	PCOS-AB (n=292)	PCOS-L (n=266)	*Z/t*	*P* value
Age(years)	31.53 ± 3.61	31.62 ± 3.57	0.30	0.77
BMI(kg/cm^2^)	25.63 ± 4.93	24.76 ± 4.24	-2.24	0.03^*^
AMH(ng/mL)	6.33(4.05,9.62)	6.64(4.15,9.16)	-0.62	0.53
LH/FSH	1.57 ± 0.98	1.50 ± 0.89	-0.86	0.39
SHBG(nmol/L)	37.50(23.10,66.60)	43.70(27.30,85.47)	-2.41	0.02^*^
Bio-T(pg/mL)	139.31(82.02,213.15)	106.06(73.07,159.16)	-4.39	<0.01^**^
A2(ng/mL)	1.65 ± 0.79	1.43 ± 0.87	-3.05	<0.01^**^
17-OHP(ng/mL)	0.80 ± 1.32	0.68 ± 0.86	-1.31	0.19
DHT(ng/mL)	0.17(0.11,0.22)	0.17(0.10,0.22)	0.75	0.46
FT(pg/mL)	5.55(3.26,8.31)	4.26(2.90,6.32)	-4.17	<0.01^**^
TT(nmol/L)	1.20(0.82,1.70)	1.06(0.73,1.36)	-3.32	<0.01^**^
FAI	0.03(0.02,0.05)	0.02(0.013,0.037)	-4.11	<0.01^**^
DHEAS(ng/mL)	1866.20(1223.55,2581.69)	1770.39(1265.96,2338.46)	-1.94	0.05
DHEA(ng/mL)	3.00(1.75,4.93)	3.12(1.89,4.73)	-0.11	0.91
HOMA-IR	3.17(2.06,4.38)	2.72(1.74,3.68)	-3.29	<0.01^**^
HCY(umol/L)	8.20(7.02,10.00)	6.70(5.97,7.60)	-10.04	<0.01^**^
PLT(*10^9^/L)	283.20 ± 59.64	263.61 ± 60.77	-3.84	<0.01^**^

^*^P<0.05; ^**^P<0.01; PCOS-L, PCOS with live birth; PCOS-AB, PCOS with spontaneous abortion group.

#### 3.3.2 Correlation analysis of endocrine and metabolic parameters in patients with live birth and spontaneous abortion

**Figure 3:**
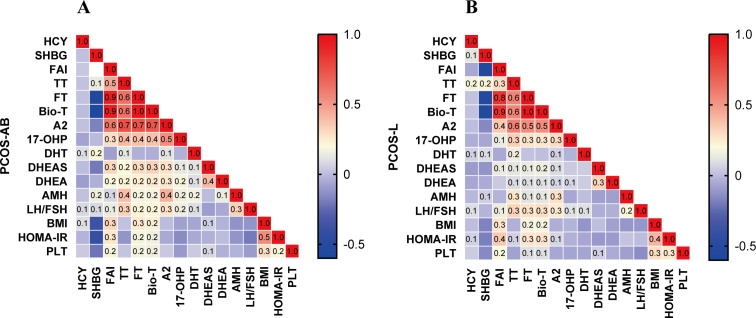
Correlation heatmap of endocrine and metabolic indices in patients with PCOS-AB and PCOS-L. **(A)** PCOS-AB,Spontaneous abortion group; **(B)** PCOS-L, Live birth group. Values in cells represent Pearson’s correlation coefficients (*r*). The color bar indicates the strength and direction of correlation: red denotes positive correlations, blue denotes negative correlations, and color intensity corresponds to the absolute magnitude of *r*.

To further investigate the correlations between HCY and androgens in patients with live birth versus spontaneous abortion, Spearman correlation analysis was performed. As shown in **Figure 3**, strong positive correlations within the androgen metabolic pathway were observed in both groups, suggesting that the intrinsic interrelationships among androgen parameters are not influenced by reproductive outcomes. In patients with spontaneous abortion, no significant correlations were observed between HCY and any endocrine or metabolic parameters (all P > 0.05) (**Figure 3A**). In contrast, patients with live birth demonstrated significant positive correlations between HCY and both SHBG and TT (P < 0.05) (**Figure 3B**). A significant between-group difference was observed in the correlation coefficient for HCY and TT (P < 0.05) (**Supplementary Table S2**).

#### 3.3.3 Multivariable linear regression analysis of HCY and androgens in patients with live birth and spontaneous abortion

In multivariable linear regression models adjusted for age, BMI, AMH, LH/FSH ratio, platelet count, and HOMA-IR, HCY was significantly and negatively associated with DHEA in patients with spontaneous abortion (P < 0.05). Specifically, each one-unit increase in DHEA was associated with a 0.162-unit decrease in HCY. In patients with live birth, HCY was significantly and positively associated with TT (P < 0.01), with each one-unit increase in TT corresponding to a 0.298-unit increase in HCY (**Table 4**).

**Table 4:** Multivariable linear regression analysis of HCY and androgens in PCOS-AB and PCOS-L groups.

Variables	PCOS-AB		PCOS-L	
	*β*	*P*	*β*	*P*
SHBG	-0.06	0.40	0.03	0.67
Bio-T	-0.00	0.99	0.12	0.20
A2	-0.06	0.56	-0.11	0.18
17-OHP	-0.07	0.35	-0.11	0.11
DHT	0.06	0.31	0.02	0.73
FT	0.02	0.82	0.12	0.19
TT	0.15	0.10	0.30	<0.01^**^
FAI	0.06	0.49	-0.04	0.65
DHEAS	0.01	0.87	-0.11	0.11
DHEA	-0.16	0.02^*^	-0.04	0.54

^*^P<0.05; ^**^P<0.01; PCOS-L, PCOS with live birth; PCOS-AB, PCOS with spontaneous abortion group. The coefficients of non-collinear variables were derived from the model including TT. These coefficients showed minimal variation across the four models, and the consistency of P-values remained unchanged, indicating model robustness.

## 4 Discussion

This study analyzed the association patterns between HCY and androgens in PCOS patients across different reproductive outcomes. Our findings revealed that the relationship between HCY and androgens is not static but undergoes complex transitions depending on reproductive status and pregnancy outcomes.

This study demonstrated significant differences in baseline characteristics between infertile PCOS patients and those who achieved pregnancy. Compared with the pregnancy group, the infertility group exhibited more pronounced metabolic and endocrine disturbances, including higher BMI, AMH, LH/FSH ratio, HOMA-IR, androgen levels (TT, Bio-T, A2, DHT, FT, FAI, DHEA), platelet count, and HCY concentrations, along with lower SHBG levels. These baseline disparities themselves represent potential pathological underpinnings of infertility. Testosterone is the most commonly used androgen marker for the clinical diagnosis of hyperandrogenism; however, its physiological and pathological effects are not solely determined by serum total testosterone levels, but rather by the bioactive fraction capable of binding androgen receptors and exerting effects in target tissues (22). In current clinical assessment of PCOS, multiple indicators—including total testosterone, free testosterone, and the free androgen index—are frequently combined to improve diagnostic accuracy (1). In the present study, we employed liquid chromatography-tandem mass spectrometry, a technique with high sensitivity and specificity (23), to comprehensively profile androgens. Our results confirmed that bioactive androgens (Bio-T, A2, DHT, FT, TT) are core drivers of reproductive dysfunction in PCOS. These androgens induce chronic low-grade ovarian inflammation, trigger granulosa cell pyroptosis, impair follicular development, and promote ovarian interstitial fibrosis, leading to ovulatory dysfunction (24–26). Furthermore, elevated androgens directly act on the endometrium, inhibiting decidualization of endometrial stromal cells and interfering with embryo implantation and early pregnancy maintenance (27, 28). In PCOS patients with reproductive failure, the bioactivity of androgens may be more critical than their total concentrations.

HCY plays distinct roles in PCOS patients with different reproductive outcomes. The positive correlation between HCY and androgens is more extensive and stronger in the infertile group. As a metabolic intermediate of sulfur-containing amino acids, HCY is a well-established risk factor for vascular endothelial injury and oxidative stress (13). Elevated homocysteine exerts significant negative effects on the reproductive system across multiple dimensions, including ovulation dysfunction, diminished oocyte and embryo quality, infertility due to abnormal sperm parameters in males, and adverse pregnancy outcomes (12, 29–31). In the context of infertility, the inherent insulin resistance and hyperandrogenism of PCOS may synergistically interfere with HCY metabolism, forming a mutually reinforcing vicious cycle. On one hand, insulin resistance is not only a core feature of PCOS metabolic disturbances, but its accompanying decrease in insulin clearance and increase in secretion can directly exacerbate the development and maintenance of hyperandrogenism (10). On the other hand, elevated androgens, through direct action on their receptors in peripheral metabolic organs, drive endothelial dysfunction and metabolic imbalance, further deteriorating insulin sensitivity (32, 33). Concurrently, HHcy can promote insulin resistance by activating inflammasomes and regulating macrophage polarization (34). Furthermore, HHcy can exacerbate androgen accumulation by inhibiting aromatase activity and reducing the conversion of androgens to estrogens (34, 35). Thus, a complex positive regulatory network forms among hyperandrogenism, insulin resistance, and HHcy in PCOS patients, collectively exacerbating endocrine and metabolic disturbances and ultimately leading to infertility. This study performed stratified measurements of androgen indicators: TT reflects overall androgen levels, FT represents the bioactive unbound fraction, and Bio-T includes both free testosterone and the fraction loosely bound to albumin; the latter two better reflect actual androgenic effects. Notably, after adjusting for confounders, HCY showed significant linear positive correlations with TT, Bio-T, and FT in the infertile PCOS group, while in the pregnant group, HCY was only significantly positively correlated with TT and negatively correlated with DHEA. This discrepancy suggests that the complex regulatory network between HCY and androgens tends to moderate in the pregnant PCOS group. During normal pregnancy, maternal HCY levels physiologically decrease (36),the placenta becomes the dominant endocrine organ, and ovarian-derived androgen synthesis is significantly reduced (37). Women with PCOS who achieve successful pregnancy inherently have lower insulin resistance, androgen levels, and HCY levels preconception, suggesting they are in a state of milder metabolic disturbance. High HCY can exacerbate androgen accumulation, while androgens can downregulate key enzymes involved in HCY metabolism, thereby interfering with its homeostasis. Therefore, the milder degree of endocrine and metabolic disturbances in the pregnant group weakens the driving force of the “HCY-androgen” vicious cycle.

To further observe the changes in the correlation patterns between HCY and androgens among PCOS patients with different pregnancy outcomes, we conducted a subgroup analysis of pregnant patients, excluding cases of non-spontaneous abortion, and compared the correlations between HCY and androgens in those who had live births and those who had spontaneous abortions. The results indicated that the abortion group exhibited more pronounced insulin resistance and hyperandrogenism. Multivariate linear regression analysis revealed a significant linear positive correlation between HCY and TT in the live birth group, whereas this correlation disappeared in the abortion group, despite significantly elevated HCY levels. The complex synergistic relationship between androgens and HCY impairs reproductive outcomes at multiple levels. Elevated androgens are a key pathological factor in decreased endometrial receptivity in PCOS. Elevated HCY can exacerbate androgen accumulation and promote mechanisms like oxidative stress and endothelial injury, impairing vascular function and affecting uterine blood supply (38). HHcy can also directly interfere with follicular growth and disrupt endometrial receptivity, increasing the risk of implantation failure and miscarriage (39). HCY is not only a marker of endothelial dysfunction but also an important regulator affecting placental vascular remodeling during pregnancy. Methionine synthase, a key enzyme involved in HCY metabolism, converts HCY to methionine, promoting glutathione synthesis and enhancing placental antioxidant capacity (40). Elevated HCY may induce local placental microthrombus formation or lead to insufficient uterine-placental circulation perfusion (41), affecting embryonic development and maintenance (42, 43). Admittedly, HCY metabolism is influenced by multiple factors including genetics, nutrition (e.g., folate, vitamin B12), and lifestyle. Specific gene polymorphisms have been identified as potential contributors to elevated HCY and adverse pregnancy outcomes (44). Folate and vitamin B12, acting as cofactors, can lower HCY levels and improve placental vascular density and function upon supplementation (45). Although this retrospective study could not obtain these critical pieces of information, elevated HCY during pregnancy is likely not an independent causative factor but rather acts as a key node within a complex network, synergizing with other metabolic and endocrine disturbances to affect pregnancy maintenance (31).

Furthermore, this study found a significant negative correlation between homocysteine and dehydroepiandrosterone uniquely in the spontaneous abortion group, an association not observed in either the infertile or the live birth groups. DHEA is primarily synthesized and secreted by the zona reticularis of the adrenal cortex and is regulated by the hypothalamic-pituitary-adrenal axis (46). As a precursor to androgens like testosterone, DHEA homeostasis is crucial for maintaining an appropriate endocrine microenvironment in early pregnancy (47). In this study, although DHEA levels showed no significant difference between the abortion and live birth groups, the abortion group exhibited significantly higher levels of HCY and testosterone (including TT, Bio-T, FT) compared to the live birth group. This suggests that high HCY might be involved in pregnancy loss by promoting the conversion of DHEA to downstream androgens or through other pathways. Notably, despite no intergroup difference in DHEA levels, the negative correlation between HCY and DHEA within the abortion group still points to a potential impact of high HCY on adrenal steroidogenic function. Previous studies have shown that hyperhomocysteinemia can damage various tissues and cells by inducing oxidative stress and epigenetic modifications (48). CYP17A1, a key enzyme in DHEA synthesis (49), is subject to epigenetic regulation, including DNA methylation (50). Disruption of redox homeostasis can affect the expression of steroidogenesis-related genes in adrenal cortical cells (51). However, whether HCY participates in pregnancy loss by regulating the synthesis of adrenal-derived androgens or by promoting their downstream conversion requires further investigation.

This study reveals that the correlation pattern between HCY and androgens in patients with PCOS is not fixed but undergoes dynamic and complex evolution depending on reproductive status, profoundly reflecting the heterogeneity in the pathological mechanisms underlying reproductive disorders in PCOS. In the infertile PCOS population, HCY exhibits broad and strong positive correlations with multiple androgens. In pregnant patients, the association between HCY and androgens gradually weakens. Among those who achieve successful live birth, only the positive correlation between HCY and total testosterone persists, with overall androgen and HCY levels tending to moderate. In patients who experience miscarriage, HCY displays a unique linear negative correlation with DHEA. This finding generates a hypothesis for our future research: whether HCY participates in the occurrence of miscarriage by regulating adrenal-derived DHEA metabolism and its downstream pathways. This will be a direction for our subsequent investigations.

## 5 Strengths and limitations

This study has several strengths. First, androgen levels were measured using mass spectrometry, a technique internationally recommended for its high accuracy and comparability. Second, a comprehensive panel of endocrine and metabolic parameters relevant to PCOS was evaluated, including BMI, HOMA-IR, AMH, LH/FSH ratio, and a broad spectrum of androgen indicators. This provided a robust data foundation for integrative analysis of the pathophysiological features of PCOS.

Nevertheless, several limitations should be acknowledged. Due to the constraints of its retrospective design, we observed correlations between HCY and the androgen profile, but causal relationships could not be established. We were unable to completely and accurately obtain medication histories for all patients, including drugs that might influence HCY metabolism or androgen levels (e.g., metformin, letrozole) and specific protocols for pregnancy maintenance after conception; consequently, stratified analyses based on these medications were not performed. Furthermore, data on lifestyle factors, as well as levels of folate and vitamin B12—which can affect HCY levels—were not collected, and the absence of these factors may limit a comprehensive assessment of HCY metabolic regulation. The prevalence of hypothyroidism (including subclinical hypothyroidism) and hyperprolactinemia is relatively high among women with PCOS seeking fertility. Strictly excluding patients with these conditions would have substantially reduced the sample size, potentially introducing confounding bias. Therefore, patients with hypothyroidism (including subclinical hypothyroidism) and hyperprolactinemia were not excluded from this study. As a single-center retrospective study, the representativeness of the sample and the generalizability of the findings are somewhat limited. Additionally, there were only four cases of late spontaneous abortion in this study, precluding subgroup analysis based on early versus late miscarriage. Given the potentially different pathological mechanisms underlying early and late pregnancy loss, larger-scale, multicenter prospective studies are needed for further validation.

## Data Availability

The original contributions presented in the study are included in the article/**Supplementary Material**. Further inquiries can be directed to the corresponding author.
