# Supplementary material for: Outcome-specific shifts in homocysteine-androgen correlations in polycystic ovary syndrome
Source: Front Endocrinol (Lausanne). 2026 Apr 7;17:1809890. doi: 10.3389/fendo.2026.1809890 (PMC13096034; doi:10.3389/fendo.2026.1809890)
Supplement: Supplementary file 1 [file Table1.doc]

| **S 1. Comparison of HCY-Androgen Correlations between PCOS-P and PCOS-I Groups** | | | | | | | | | | |
| --- | --- | --- | --- | --- | --- | --- | --- | --- | --- | --- |
| Groups/r | SHBG | Bio-T | A2 | 17-OHP | DHT | FT | TT | FAI | DHEAS | DHEA |
| PCOS-P (n=573) | 0.01 | 0.08 | 0.09 | -0.00 | 0.06 | 0.09 | 0.15 | 0.08 | 0.01 | -0.05 |
| PCOS-I  (n=302) | -0.12 | 0.23 | 0.21 | -0.07 | 0.10 | 0.23 | 0.20 | 0.22 | 0.16 | 0.06 |
| *Z* | 1.68 | -2.24 | -1.97 | 0.96 | -0.66 | -2.16 | -0.83 | -2.11 | -2.31 | -1.96 |
| *P* | 0.09 | 0.03^*^ | 0.05^*^ | 0.34 | 0.51 | 0.03^*^ | 0.41 | 0.04^*^ | 0.02^*^ | 0.05 |
| ^*^*P*＜0.05；PCOS-P,PCOS with Pregnancy；PCOS-I,PCOS with Infertility. | | | | | | | | | | |

| **S 2. Comparison of HCY-Androgen Correlations between PCOS-AB and PCOS-L Groups** | | | | | | | | | | |
| --- | --- | --- | --- | --- | --- | --- | --- | --- | --- | --- |
| Groups/r | SHBG | Bio-T | A2 | 17-OHP | DHT | FT | TT | FAI | DHEAS | DHEA |
| PCOS-AB  (n=292) | 0.01 | -0.00 | 0.01 | -0.07 | 0.09 | 0.02 | 0.02 | 0.03 | 0.00 | -0.09 |
| PCOS-L  (n=266) | 0.13 | 0.00 | 0.02 | -0.04 | 0.01 | 0.01 | 0.22 | -0.03 | -0.08 | -0.03 |
| *Z* | -1.37 | -0.02 | -0.08 | -0.42 | 1.00 | 0.10 | -2.34 | 0.72 | 0.88 | -0.70 |
| *P* | 0.17 | 0.98 | 0.94 | 0.67 | 0.32 | 0.92 | 0.02^*^ | 0.47 | 0.38 | 0.48 |
| ^*^*P*＜0.05；PCOS-L ,PCOS with live birth; PCOS-AB,PCOS with spontaneous abortion group. | | | | | | | | | | |
